# Characterization of fructooligosaccharide metabolism and fructooligosaccharide-degrading enzymes in human commensal butyrate producers

**DOI:** 10.1080/19490976.2020.1869503

**Published:** 2021-01-13

**Authors:** Hiroki Tanno, Tadashi Fujii, Katsuaki Hirano, Shintaro Maeno, Takashi Tonozuka, Mitsuo Sakamoto, Moriya Ohkuma, Takumi Tochio, Akihito Endo

**Affiliations:** a Department of Food, Aroma and Cosmetic Chemistry, Faculty of Bioindustry, Tokyo University of Agriculture, Hokkaido, Japan; b B Food Science Co., Ltd., Aichi, Japan; c Department of Applied Biological Science, Tokyo University of Agriculture and Technology, Tokyo, Japan; d PRIME, Japan Agency for Medical Research and Development (AMED), Ibaraki, Japan; e Microbe Division/Japan Collection of Microorganisms, RIKEN BioResource Research Center, Ibaraki, Japan

**Keywords:** Butyrate producing bacteria, GH32, fructooligosaccharide, kestose, nystose, genome

## Abstract

Butyrate produced by gut microbiota has multiple beneficial effects on host health, and oligosaccharides derived from host diets and glycans originating from host mucus are major sources of its production. A significant reduction of butyrate-producing bacteria has been reported in patients with inflammatory bowel diseases and colorectal cancers. Although gut butyrate levels are important for host health, oligosaccharide metabolic properties in butyrate producers are poorly characterized. We studied the metabolic properties of fructooligosaccharides (FOSs) and other prebiotic oligosaccharides (i.e. raffinose and xylooligosaccharides; XOSs) in gut butyrate producers. 1-Kestose (kestose) and nystose, FOSs with degrees of polymerization of 3 and 4, respectively, were also included. Fourteen species of butyrate producers were divided into four groups based on their oligosaccharide metabolic properties, which are group A (two species) metabolizing all oligosaccharides tested, group F (four species) metabolizing FOSs but not raffinose and XOSs, group XR (four species) metabolizing XOSs and/or raffinose but not FOSs, and group N (four species) metabolizing none of the oligosaccharides tested. Species assigned to groups A and XR are rich glycoside hydrolase (GH) holders, whereas those in groups F and N are the opposite. In total, 17 enzymes assigned to GH32 were observed in nine of the 14 butyrate producers tested, and species that metabolized FOSs had at least one active GH32 enzyme. The GH32 enzymes were divided into four clusters by phylogenetic analysis. Heterologous gene expression analysis revealed that the GH32 enzymes in each cluster had similar FOS degradation properties within clusters, which may be linked to the conservation/substitution of amino acids to bind with substrates in GH32 enzymes. This study provides important knowledge to understand the impact of FOS supplementation on the activation of gut butyrate producers.

**Abbreviations:** SCFA, short chain fatty acid; FOS, fructooligosaccharide; XOS, xylooligosaccharide; CAZy, Carbohydrate Active Enzymes; CBM, carbohydrate-binding module; PUL, polysaccharide utilization locus; S6PH sucrose-6-phosphate hydrolase.

## Introduction

The human gut microbiota plays essential roles in host health, including food digestion, immunological homeostasis, protection against colonization by pathogens, and the extraction and storage of energy from food components. Recent studies also suggested that the impact of the gut microbiota on the host is not restricted to gut health, but also includes brain development and behavior (gut-brain axis),^1^ lung health (gut-lung axis),^2^ nephrolithiasis and renal phosphorus homeostasis (gut-kidney or gut-renal axis),^3,4^ physical exercise (gut-joint axis),^5^ and cardiovascular health (gut-heart axis).^6^ The microbiota excretes many metabolites, which are sometimes essential for host homeostasis. Short chain fatty acids (SCFAs) are among the essential metabolites. Most gut microbes produce SCFAs and/or organic acids in a species- or sometimes strain-specific manner,^7^ and these have marked impacts on host health. Butyrate, one of the SCFAs produced by the gut microbiota, is the major energy source for epithelial cells in the distal colon,^8^ induces differentiation of the colonic regulatory T cells,^9^ and functions as an inhibitor of histone deacetylase in the host.^10^ These activities are essential for the documented beneficial properties of butyrate, including anti-inflammation,^11^ gut immune homeostasis,^9^ inhibition of proliferation, and induction of apoptosis of colorectal cancer cells.^12^

When compared with other SCFAs or lactate, relatively limited bacteria are responsible for butyrate production in the human gut microbiota. They are mainly specific species in the *Clostridium* clusters IV and XIVa.^13^ Significant reduction of the gut butyrate producers has been reported in patients with Crohn’s disease,^14^ ulcerative colitis,^15^ diabetes,^16^ colorectal cancer,^17^ and infantile food allergy^18^ when compared with healthy subjects. These microbes usually produce butyrate by metabolizing carbohydrates through butyryl-CoA:acetate CoA-transferase (encoded by the *but* gene) or butyrate kinase (encoded by the *buk* gene), but some use lactate and acetate as sources for its production.^19,20^ Different animal species possess different profiles of gut butyrate producers and diet habits impact the profiles.^21^ The human gut butyrate producers are highly sensitive to oxygen and the use of organisms as probiotics is not easily applicable due to the difficulty in maintaining their viability. The application of prebiotic oligosaccharides is thus a reasonable tool for the proliferation of the gut organisms. Short-chain fructooligosaccharide (FOS) is one of the well commercialized and investigated prebiotics, and increased butyrate production and/or proliferation of butyrate-producing microbes has been reported in healthy adults, infants with atopic dermatitis, and several animals after the administration of short-chain FOS.^22–25^ Commercialized short-chain FOS is generally a mixture of FOSs with a degree of polymerization (DP) of 3 and 4, i.e. 1-kestose (kestose) and nystose, respectively, with trace DP5 FOS, fructosylnystose.^26^ The former study reported that the DP of FOS markedly affects the growth of bifidobacteria and lactic acid bacteria.^7,26^ Moreover, the DP significantly affected the growth of a human commensal butyrate producer, *Anaerostipes caccae*;^7^ however, FOS metabolic properties of most human commensal butyrate producers focusing on DP have been poorly characterized. FOSs are generally hydrolyzed by glycoside hydrolase (GH) family 32 enzymes,^27^ and the resultant fructose and glucose are metabolized through indigenous metabolic pathways in each microbe.^28,29^ GH32 enzymes thus play a key role in FOS metabolism, but few studies to date have characterized the prevalence and activity of GH32 enzymes in butyrate-producing gut microbes.

In the present study, FOS metabolic properties in human commensal butyrate-producing bacteria, taxonomically classified into *Clostridium* clusters IV and XIVa and the genus *Butyricimonas* (phylum Bacteroidetes), were characterized. A xylooligosaccharide mixture (XOSs) and raffinose, which are used as bifidogenic prebiotics,^30,31^ were also included in this study. Genomes of the butyrate producers were used to study the prevalence of GH32 enzymes. Activities of the GH32 enzymes were characterized by the *Escherichia coli* heterologous expression system.

## Results

### Oligosaccharide metabolic properties

**Figure 1.**
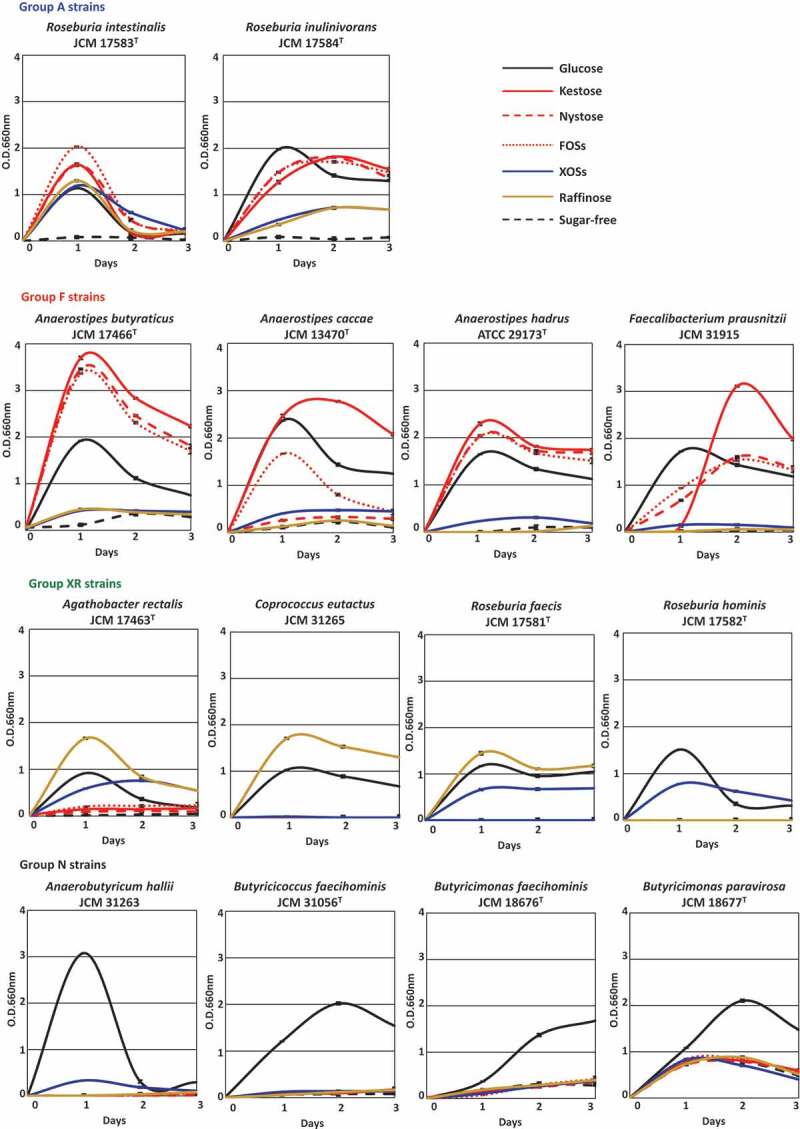
Growth of 14 butyrate-producing bacteria on several oligosaccharides. Growth was monitored at 660 nm in basal YCFA broth supplemented with 0.5% (w/v) oligosaccharides, including kestose, nystose, fructooligosaccharide mixture (FOSs), xylooligosaccharide mixture (XOSs), and raffinose, and glucose and sugar-free broths were included as controls. Data were obtained every 24 h until 72 h. Data plotted in graphs are the means ± standard deviations of triplicates of strains

**Figure 2.**
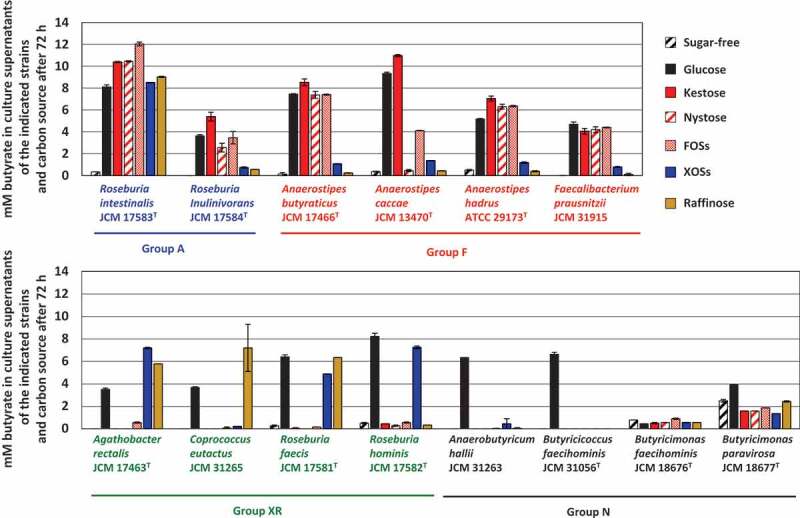
Concentration of butyrate in culture supernatants of 14 butyrate producers in the presence of each oligosaccharide (0.5%, w/v) after incubation of 72 h. Bars and error bars indicate means and standard deviations from triplicates, respectively. Groups A, F, XR, and N correspond to the metabolic groups shown in Figure 1

Fourteen butyrate-producing bacteria used in the present study are shown in Table 1. They had different oligosaccharide metabolic properties. *Roseburia intestinalis* and *Roseburia inulinivorans* grew with all oligosaccharides tested (hereafter group A), although growth levels were slightly different among oligosaccharides for *R. inulinivorans* (Figure 1). *Faecalibacterium prausnitzii* and three species of the genus *Anaerostipes* actively grew on FOS-type oligosaccharides, i.e. kestose, nystose, and FOSs (hereafter group F), but growth of *A. caccae* was not observed with nystose (Figure 1). Their growth on raffinose or XOSs was similar level to that on sugar-free medium. On the other hand, *Agathobacter rectalis, Coprococcus eutactus, Roseburia faecis*, and *Roseburia hominis* did not metabolize the FOS-type oligosaccharides, and instead metabolized XOSs and/or raffinose (hereafter group XR). *Anaerobutyricum hallii, Butyricicoccus faecihominis*, and two species of *Butyricimonas* did not metabolize any of the oligosaccharides tested (hereafter group N). Levels of butyrate production from the metabolism of oligosaccharides after 72 h of incubation were generally consistent with their growth (Figure 2), except that marked butyrate production was not noted by *Butyricimonas faecihominis*.

**Table 1.** Butyrate-producing bacteria used in the present study

Phylogenetic group	Bacterial strains	Metabolic group*	Genomic accession number	Genome level	Size (Mb)	Number of CDSs
*Clostridium* cluster IV	*Faecalibacterium prausnitzii* JCM 31915	F	GCA_002734145.1	Complete	3.11	2790
*Clostridium* cluster IV	*Butyricicoccus faecihominis* JCM 31056 ^T^	N	BLYJ01000000	Draft	3.03	2857
*Clostridium* cluster XIVa	*Agathobacter rectalis* JCM 17463 ^T^	XR	GCA_000020605.1	Complete	3.45	3161
*Clostridium* cluster XIVa	*Anaerobutyricum hallii* JCM 31263	N	BLYK01000000	Draft	3.74	3261
*Clostridium* cluster XIVa	*Anaerostipes butyraticus* JCM 17466 ^T^	F	BLYI01000000	Draft	3.17	3183
*Clostridium* cluster XIVa	*Anaerostipes caccae* JCM 13470 ^T^	F	GCA_000154305.1	Draft	3.61	3347
*Clostridium* cluster XIVa	*Anaerostipes hadrus* ATCC 29173 ^T^	F	GCA_000332875.2	Draft	2.77	2589
*Clostridium* cluster XIVa	*Coprococcus eutactus* JCM 31265	XR	BLYL01000000	Draft	3.25	2865
*Clostridium* cluster XIVa	*Roseburia faecis* JCM 17581 ^T^	XR	GCA_001406815.1	Draft	3.33	3046
*Clostridium* cluster XIVa	*Roseburia hominis* JCM 17582 ^T^	XR	GCA_000225345.1	Complete	3.59	3148
*Clostridium* cluster XIVa	*Roseburia intestinalis* JCM 17583 ^T^	A	GCA_900537995.1	Complete	4.49	3928
*Clostridium* cluster XIVa	*Roseburia inulinivorans* JCM 17584 ^T^	A	GCA_000174195.1	Draft	4.05	3559
Bacteroidetes	*Butyricimonas faecihominis* JCM 18676 ^T^	N	2830045573**	Draft	4.79	4004
Bacteroidetes	*Butyricimonas paravirosa* JCM 18677 ^T^	N	JAATLI010000000	Draft	5.53	4394

*Determined based on oligosaccharide metabolic properties in Figure 1

**Obtained from the Integrated Microbial Genomes (IMG) database at the Department of Energy Joint Genome Institute (http://genome.jgi.doe.gov/)

**Figure 3.**
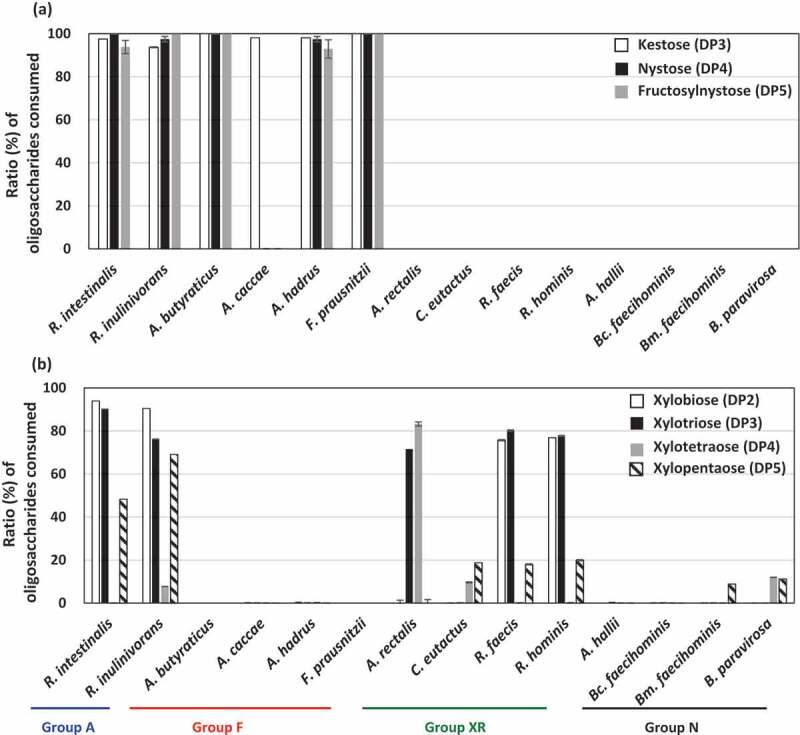
Ratio of each oligosaccharide consumed (%) after metabolism of FOSs (a) and XOSs (b) in butyrate producers. Ratios of oligosaccharides consumed were measured in culture supernatants of YCFA broth supplemented with 0.5% (w/v) FOSs (a) or 0.5% (w/v) XOSs (b) after incubation for 72 h. Kestose, nystose, and fructosylnystose were the major components of FOSs (>95% components in total), whereas xylobiose, xylotriose, xylotetraose, and xylopentaose were the major components of XOSs (>90% components in total). Bars and error bars indicate means and standard deviations from triplicates, respectively. Bars are absent when the strains did not metabolize the oligosaccharides. Groups A, F, XR, and N correspond to the metabolic groups shown in Figure 1. *Bc. faecihominis, Butyricicoccus faecihominis; Bm. faecihominis, Butyricimonas faecihominis.*

As FOSs and XOSs are mixtures of different DP oligosaccharides, oligosaccharide metabolic profiles were assessed by measuring the remaining oligosaccharides after culturing. Members in groups A and F metabolized all oligosaccharides in FOSs well, except *A. caccae*, which completely consumed kestose but left nystose and fructosylnystose (Figure 3(a)). Members in groups XR and N did not consume oligosaccharides in FOSs. Regarding XOSs, strains in group A consumed DP2, DP3, and DP5 oligosaccharides but left DP4 oligosaccharides (Figure 3(b)). Of the group XR strains, *R. faecis* and *R. hominis* mainly consumed DP2 and DP3 oligosaccharides, but *A. rectalis* consumed DP3 and DP4 oligosaccharides and left DP2 oligosaccharide. This suggests that even if butyrate producers grow on XOSs, metabolized oligosaccharides vary among the species. Species that did not actively grow on XOSs (Figure 1) did not exhibit marked consumption of oligosaccharides in XOSs.

### Identification of GH family proteins

**Figure 4.**
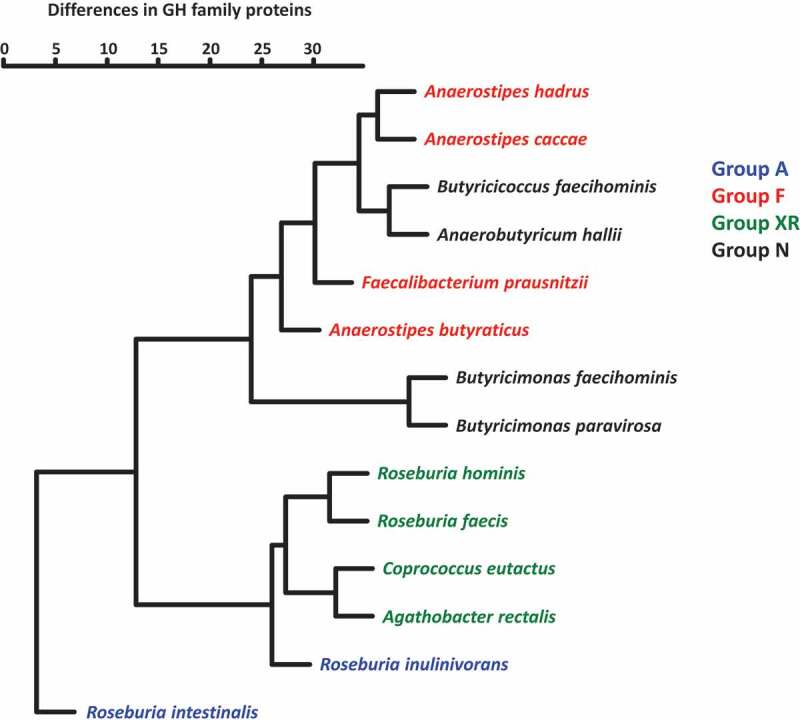
Hierarchical clustering of 14 butyrate-producing bacteria based on the presence of GH family proteins. Numbers of estimated proteins in each GH family were used to prepare a dendrogram using the hclust function with the Ward.D2 algorithm in the R package (version 3.6.2)

Complete or draft genomes of the 14 strains (Table 1) were used to search for GH family proteins using dbCAN2 in the Carbohydrate Active Enzymes (CAZy) database. In total, 61 GH families were found in genomes of the butyrate producers and strains often possessed multiple proteins in a single GH family (Table 2 and Supplemental Table S1). Members in groups A and XR possessed 53–124 and 50–66 GH family proteins, respectively, and these numbers were much larger than those of members in groups F (18–32 proteins) and N (10–25 proteins). Of the 61 GH families found, GH3 and GH13 were conserved in all strains tested, whereas 13 families were unique to a specific strain (Table 2). GH57, GH63, GH84, GH92, and GH109 proteins were only found in *Butyricimonas*. One or two proteins assigned to GH4, which were annotated as 6-phospho-α-glucosidase and 6-phospho-β-glucosidase, were common in *Anaerostipes* spp. (Supplemental Table S1), but rare in other strains. GH5, GH8, GH31, GH42, GH43, GH51, and GH94 are unique GH families in groups A and XR with few exceptions (Table 2). All proteins assigned to GH8 found in groups A and XR were described as possible XOS degradation proteins (Supplemental Table S1). GH32 proteins, which are possibly involved in FOS degradation, were found in all members of groups A and F, and three of the four strains in group XR but not in group N. Hierarchical clustering analysis based on the number of proteins in each GH family produced two major clusters (Figure 4). One of the two consisted of members in groups F and N, and the other was composed of group XR and *R. inulinivorans. Roseburia intestinalis* was distantly positioned from other species, which was due to the presence of a larger number of GH proteins and several unique proteins. The phylogenetic position of the species was not related to this clustering.

**Table 2.** Numbers of GH family proteins found in each butyrate producing bacteria

### Phylogenetic analysis and activities of GH32 proteins

**Figure 5.**
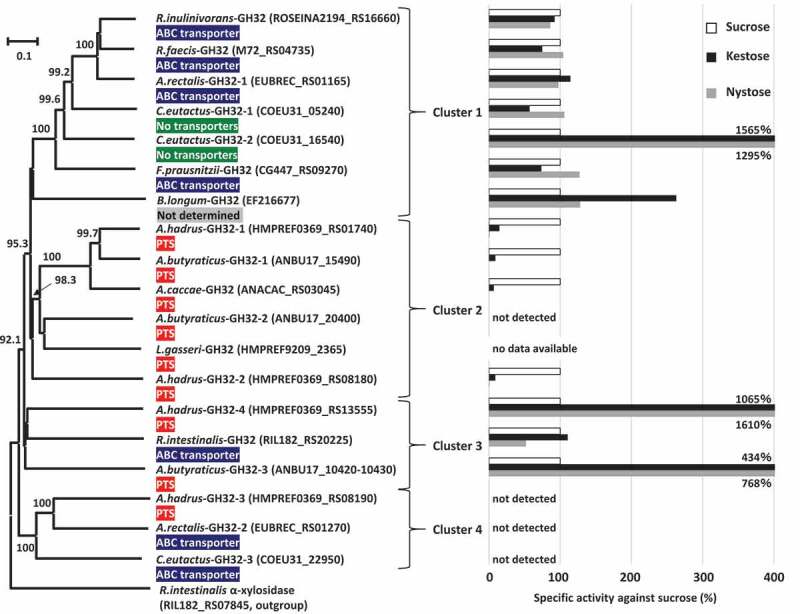
Phylogenetic relationships of GH32 enzymes found in butyrate-producing bacteria, and their relative specific activities with kestose and nystose against sucrose. Locus tags of each protein are shown in parenthesis and transporters adjacent to each GH32 protein are supplied. Extracellular GH32 enzymes (*A.hadrus*-GH32-4 and *A.butyraticus*-GH32-3) were expressed using a surface display system on *E. coli* cells, and whole cells of the transformed *E. coli* were included to assess the activities of the GH32 enzymes on sucrose, kestose, and nystose. For intracellular GH32 enzymes, cell-free extracts prepared from the transformed *E. coli* strains were used to assess the activities. Relative specific activities (%) of recombinant GH32 enzymes with kestose and nystose against sucrose are shown. For *A.butyraticus*-GH32-3, specific activities by the fused enzyme (ANBU17_10420 and ANBU17_10430) are shown, and those by the partial *A.butyraticus*-GH32-3 (ANBU17_10420) were unable to be assessed because of the lack of activity with sucrose. *B.longum*-GH32 and *L.gasseri*-GH32, originating from *B. longum* KN29.1 and *L. gasseri* 224–1, respectively, were included as reference proteins, and a possible transporter for *B.longum*-GH32 was unable to be identified due to the unavailability of genome data for *B. longum* KN29.1. Specific activities for *B.longum*-GH32 and *A.caccae*-GH32 were obtained from previous reports.^32,33^ α-Xylosidase in *Roseburia intestinalis* (RIL182_RS07845) was used as an outgroup. Bootstrap percentages above 70% are given at branching points

Phylogenetic analysis was conducted based on amino acid sequences of the 17 proteins assigned to GH32 found in the butyrate producers, and two reference GH32 enzymes originating from *Bifidobacterium longum* strain KN29.1 and *Lactobacillus gasseri* strain 224–1, whose crystal structures have been determined, were also included in this analysis. The phylogenetic tree produced four major clusters (Figure 5). Cluster 1 included *A.rectalis*-GH32-1, *C.eutactus*-GH32-1, *C.eutactus*-GH32-2, *F.prausnitzii*-GH32, *R.faecis*-GH32, *R.inulinivorans*-GH32, and a reference, *B.longum*-GH32. The genes encoding these GH32 proteins were adjacent to genes encoding ABC transporters, except that possible transporters were not found adjacent to *C.eutactus*-GH32-1 and *C.eutactus*-GH32-2. Genes encoding the two proteins in *C. eutactus* were adjacent to genes encoding conjugal transfer protein or integrase TN1549-like. The genome of the strain *B. longum* KN29.1 has not been published and a possible transporter was unable to be identified for *B.longum*-GH32. Cluster 2 consisted of five GH32 proteins found in three species of *Anaerostipes* and a reference enzyme, *L.gasseri-*GH32. All genes encoding these proteins were located adjacent to genes encoding the phosphotransferase system (PTS). Cluster 3 included *R.intestinalis*-GH32, *A.butyraticus*-GH32-3, and *A.hadrus*-GH32-4, and the first was adjacent to ABC transporter and the latter two were adjacent to the PTS. Cluster 4 contained GH32 proteins of *A.hadrus*-GH32-3, *A.rectalis*-GH32-2, and *C.eutactus*-GH32-3, and the first was adjacent to the PTS and the latter two were adjacent to ABC transporters. Of these 17 GH32 proteins found in butyrate producers, signal sequences and family 66 carbohydrate-binding modules (CBM) were identified only from *A.butyraticus*-GH32-3 and *A.hadrus*-GH32-4 in cluster 3. The gene encoding *A.butyraticus*-GH32-3 (4140 bp in total) contains a stop codon at the 200^th^ codon, and the gene is separated into two open reading frames (Locus_Tag ANBU17_10420 and ANBU17_10430). One encodes a 199-amino acid residue protein containing 32 amino acid residues of a signal sequence (Locus_Tag ANBU17_10430), and the other produces a 1179-amino acid residue protein with a catalytic domain (ANBU17_10420). The presence of the stop codon was re-confirmed by Sanger sequencing (data not shown).

**Figure 6.**
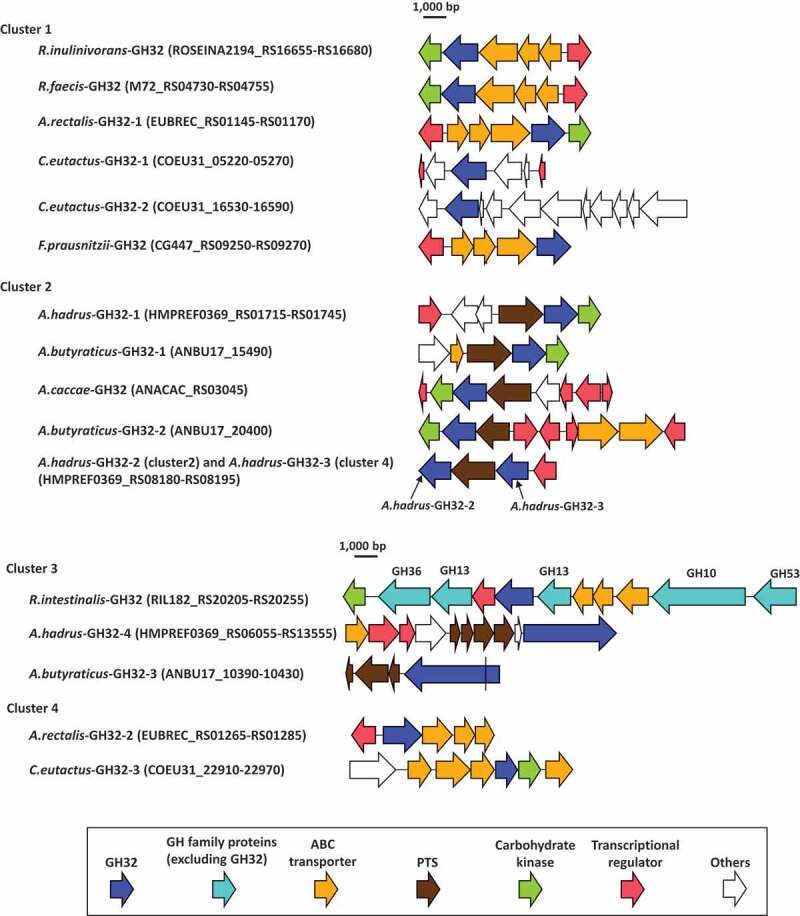
Gene arrangements surrounding GH32 proteins found in butyrate-producing bacteria. Range of locus tags of the genes shown are indicated in parenthesis. *A.hadrus*-GH32-2 of cluster 2 and *A.hadrus*-GH32-3 of cluster 4 formed a single PUL. The gene encoding *A.butyraticus*-GH32-3 contains a stop codon (shown as a black line in the gene) and is separated into two coding sequences based on genomic data. The presence of the stop codon was re-confirmed by Sanger-sequencing

In addition to the genes encoding carbohydrate transport function proteins, transcriptional regulators and carbohydrate kinases were also located with most of the genes encoding GH32 proteins and formed polysaccharide utilization loci (PULs, Figure 6), as described previously.^34,35^ The PUL of *R.intestinalis*-GH32 contains five more GH family proteins, including GH10, two GH13 proteins, GH36, and GH53, of which the GH10 and GH53 proteins are extracellular enzymes (Supplemental Table S1). Genes encoding *A.hadrus*-GH32-2 and *A.hadrus*-GH32-3 formed a single PUL, and sandwiched a gene encoding the PTS. Carbohydrate kinases were located with nine of 17 GH32 proteins and transcriptional regulators were with 12 of the GH32 proteins.

Genes encoding the GH32 enzymes found in butyrate producers were cloned into a plasmid and transferred to *Escherichia coli*. For intracellular GH32 enzymes, cell-free extracts prepared from the transformed *E. coli* strains were used to study the activities of the GH32 enzymes on sucrose, kestose, and nystose. Extracellular GH32 enzymes were expressed using a surface display system on *E. coli* cells, and whole cells of the transformed *E. coli* were included to assess the activity. Activities of *A.butyraticus*-GH32-3 containing a stop codon were evaluated by preparation of the two recombinants, one intracellularly expressing the catalytic domain of *A.butyraticus*-GH32-3 (Locus_Tag ANBU17_10420) and the another expressing the entire *A.butyraticus*-GH32-3 (Locus_Tag ANBU17_10420 and ANBU17_10430) fused by amino acid replacement of the stop codon with a glutamine codon using the surface display system. The cell-free extracts of *E. coli* harboring pET28a plasmid or intact *E. coli* cells harboring pCDF-PgsA plasmid degraded none of the tested substrates. Cell-free extracts containing any one of the six GH32 enzymes in cluster 1 degraded all tested substrates well (Figure 5). These enzymes generally degraded the three substrates at similar levels, except that degradation activity in *C.eutactus*-GH32-2 was more than 10-times higher with kestose and nystose than with sucrose. Cell-free extracts containing three (*A.butyraticus*-GH32-1, *A.hadrus*-GH32-1, and *A.hadrus*-GH32-2) of the five GH32 enzymes in cluster 2 actively degraded sucrose, but exhibited lower relative degradation activity with kestose (ranging from 9 to 15%) than with sucrose. Nystose was not degraded (relative degradation activity below 1%). These characteristics are consistent with those of *A.caccae*-GH32 reported previously.^32^ Degradation of the three substrates was not detected by cell-free extracts containing *A.butyraticus*-GH32-2. In cluster 3, cell-free extracts containing *R.intestinalis*-GH32 and *E. coli* cells displaying extracellular *A.hadrus*-GH32-4 degraded the three substrates well, and the latter degraded kestose and nystose markedly more than sucrose (relative degradation activity over 1,000%). Cell-free extracts containing a partial *A.butyraticus*-GH32-3 excluding the N-terminal sequence and signal sequence region (expressing LocusTag ANBU17_10420) degraded kestose and nystose but not sucrose, whereas *E. coli* cells with the fused whole *A.butyraticus*-GH32-3 actively degraded all three substrates. Kestose and nystose were degraded more than sucrose by the fused *A.butyraticus*-GH32-3 (relative degradation activity ranging from 434 to 768%). Cell-free extracts containing GH32 enzymes in cluster 4 did not degrade sucrose, kestose, or nystose. Arabinan, inulin, and levan were also included in this assay, but none of these newly included substrates were degraded by the enzymes in cluster 4.

Eight amino acid residues each in *B.longum*-GH32 (β-fructofuranosidase) and *L.gasseri*-GH32 (sucrose-6-phosphate hydrolase, S6PH) form hydrogen bonds with β-fructofuranose at subsite −1. Among the eight amino acid residues, Trp78 in *B.longum*-GH32 is substituted by His71 in *L.gasseri*-GH32 and Lys74 in *L.gasseri*-GH32 is substituted by Met81 in *B.longum*-GH32 (Supplemental Fig. S1, Table 3), resulting in the nine total amino acid residues listed in Table 3 being essential for the binding to β-fructofuranose at subsite −1. The corresponding amino acid residues of the GH32 enzymes in butyrate producers were determined based on multiple sequence alignment. The eight amino acid residues in *B.longum*-GH32 were fully conserved in GH32 enzymes belonging to cluster 1, but one (Trp78), one (Ser114), and two/three (Asn53, Gln70, and Trp78) amino acid substitution(s) were found in clusters 2, 3, and 4, respectively (Supplemental Fig. S1, Table 3). GH32 enzymes of cluster 2 had no amino acid substitutions when compared with the eight amino acid residues in *L.gasseri*-GH32, and one (Lys74), two (Lys74 and Ser107), and two/three (Asn46, Gln63, and Lys74) amino acid substitution(s) were found in clusters 1, 3, and 4, respectively. These substitutions were not noted in catalytic triads or the RDP motif. The NDPNG motif, which is characteristic of GH32 enzymes,^36^ was conserved in GH32 enzymes of clusters 1, 2, and 3, but not in those of cluster 4 (Supplemental Fig. S1).

**Table 3.** Amino acid residues at positions involved in hydrogen bonds with β-fructofuranose in *B.longum*-GH32 and *L.gasseri*-GH32

*B.longum*-GH32*	*L.gasseri*-GH32**	Cluster 1 (n = 6)	Cluster 2 (n = 5)	Cluster 3 (n = 3)	Cluster 4 (N = 3)	
Asn53	Asn46	**Asn**	**Asn**	**Asn**	Gly	**NDPNG motif Catalytic triad, NDPNG motif**
Asp54	Asp47	**Asp**	**Asp**	**Asp**	**Asp**	
Gln70	Gln63	**Gln**	**Gln**	**Gln**	**Gln**, Leu	
Trp78	(His71)***	**Trp**	Gly, His	**Trp**	Gly, Ala, Arg	
(Met81)***	Lys74	Met	**Lys**	Met, Leu	Thr, Phe	
Ser114	Ser107	**Ser**	**Ser**	Thr, Ala	**Ser**	**RDP motif Catalytic triad, RDP motif Catalytic triad, ECP motif**
Arg180	Arg166	**Arg**	**Arg**	**Arg**	**Arg**	
Asp181	Asp167	**Asp**	**Asp**	**Asp**	**Asp**	
Glu235	Glu222	**Glu**	**Glu**	**Glu**	**Glu**	

Amino acids identical with those of the two reference enzymes are written in bold. Clusters 1 to 4 correspond to clusters shown in Fig. 5.

*Eight amino acid residues involved in hydrogen bonds with β-fructofuranose in *B.longum*-GH32

**Eight amino acid residues involved in hydrogen bonds with β-fructofuranose in *L.gasseri*-GH32

***(Met81) in *B.longum*-GH32 and (His71) in *L.gasseri*-GH32 are not involved in forming a hydrogen bond with β-fructofuranose but are equivalent loci to Lys74 in *L.gasseri*-GH32 and Trp78 in *B.longum*-GH32, respectively

## Discussion

Although gut butyrate levels are important for host health, oligosaccharide metabolic properties in butyrate producers are poorly characterized. Although certain oligosaccharides, e.g. human milk oligosaccharides, have multiple functions in host health,^37^ proliferation of beneficial microbes is one of the most important characteristics in dietary oligosaccharides.

Based on the growth properties in the presence of oligosaccharides, 14 butyrate producers were divided into four groups, i.e. group A, group F, group XR, and group N. FOS-type oligosaccharides were metabolized by only six strains in groups A and F, and eight of the 14 strains did not, suggesting that FOS-type oligosaccharides are an energy source for some butyrate producers. *Faecalibacterium prausnitzii*, which is the most abundant butyrate producer in the healthy human gut and produces an anti-inflammatory molecule,^38^ metabolized only FOS-type oligosaccharides among the tested oligosaccharides. *Anaerostipes* spp. exhibited a similar pattern, except that *A. caccae* metabolized kestose but not nystose. This is consistent with a previous report.^7^ This suggests that the impact of the DP of FOSs on growth is not a common characteristic in the genus *Anaerostipes*, but rather a species- or strain-specific trait. The four strains in group F possess one to four genes encoding GH32 enzymes in their genomes. Among the group F strains, *F. prausnitzii* and *A. hadrus* possess GH32 enzymes that actively degrade nystose, but GH32 enzyme in *A. caccae* (*A.caccae*-GH32) did not. This may be the reason for the different nystose metabolic properties among the strains. A previous study revealed that genes encoding *A.caccae*-GH32 and clustered PTS and fructokinase were highly transcribed in cells cultured with kestose, but poorly transcribed in cells with nystose.^32^ *Anaerostipes butyraticus*, which metabolizes nystose, possesses three GH32 enzymes. Two of the three are *A.butyraticus*-GH32-1 degrading sucrose and kestose but not nystose, and *A.butyraticus*-GH32-2, exhibiting no degradation activity in the present study. The last is *A.butyraticus*-GH32-3, containing a stop codon. Fused complete *A.butyraticus*-GH32-3 by replacement of the stop codon with a glutamine codon and partial *A.butyraticus*-GH32-3 had similar characteristics, and degraded kestose and nystose with trace or no degradation of sucrose. The partial *A.butyraticus*-GH32-3 may therefore be responsible for the degradation of nystose by this strain. Another possibility is amino acid replacement at the stop codon in *A.butyraticus*-GH32-3 by a suppressor tRNA, leading to an intact extracellular *A.butyraticus*-GH32-3 being produced in *A. butyraticus* cells, as described in other microbes.^39,40^ All four strains in group F lacked GH8 proteins, which are potentially involved in the hydrolysis of XOSs.^41^ This is consistent with the results obtained by the *in vitro* culture study. Metabolism of raffinose requires the combination of a few enzymes, including GH36 α-galactosidase, GH32 β-fructosidase/GH13 sucrose phosphorylase, and melibiose/raffinose transporters.^42–44^ GH36 α-galactosidase and GH32 β-fructosidase are present in the genomes of three of the four strains (excluding *A. hadrus*) in group F (Table 3), but these strains did not metabolize raffinose, suggesting that transporters for melibiose or raffinose are missing.

*Roseburia intestinalis* and *R. inulinivorans* in group A metabolized all oligosaccharides tested. These strains possess GH32, GH36, and GH8 proteins, which may be involved in the metabolism of FOSs, raffinose, and XOSs, respectively, except that *R. inulinivorans* lacks GH8 proteins. The reason for this discrepancy is unclear, but GH30 protein, which is a unique protein in the strain, may function in the degradation of XOS, as described for other microbes.^45,46^ *Roseburia intestinalis* and *R. inulinivorans* possess a single GH32 enzyme. The enzymes shared similar degradation activities to FOSs, but they were located in different clusters in the phylogenetic tree (Figure 5). GH32 enzyme in *R. inulinivorans* is the most well examined GH32 enzyme in gut butyrate-producing bacteria, and was previously characterized to degrade sucrose, kestose, and nystose.^27^ The enzyme also degrades inulin and is strongly induced in its presence.^27^ *Roseburia intestinalis* has the best repertoire of GH proteins and possesses 124 GH proteins. Proteins assigned to GH35, GH38, GH74, GH95, GH125, and GH148 are unique to the strain. This organism has been linked to the degradation of several non-digestible carbohydrates, including β-mannan, xylan, and dietary plant polysaccharides.^47–49^ This species also plays a role in the deacetylation of hemicellulose, which leads to efficient utilization of dietary fiber by gut microbiota.^50^ These may be an advantage in the organism to survive in the complex and competitive gut microbiota.

Four organisms in group XR did not metabolize FOS-type oligosaccharides, whereas GH32 proteins were conserved in three of the four strains, i.e. *A. rectalis, C. eutactus*, and *R. faecis*. Although two of the GH32 proteins found in this group did not exhibit degradation activity against sucrose, kestose or nystose, the three strains possessed at least one GH32 enzyme degrading the FOS-type oligosaccharides. A possible reason for these conflicting results is the inactive induction of genes encoding the GH32 enzymes in the three strains. *Bifidobacterium longum* JCM 1217 ^T^, which actively metabolizes kestose but not nystose, possesses β-fructofuranosidase, having degradation activity against both kestose and nystose, but the gene encoding β-fructofuranosidase was only transcribed in cells cultured with kestose.^32^ This should be noted because recent metagenomic approaches are sometimes included to understand the metabolic potential of microbiota without *in vitro* tests.^51^ The present study suggested that even if functional genes are present in microbes, they are sometimes unable to metabolize the substrates. Genes encoding possible transporters were not found adjacent to the genes encoding *C.eutactus*-GH32-1 and *C.eutactus*-GH32-2, which are active against FOSs. These genes were linked with genes encoding conjugal transfer protein or integrase TN1549-like, suggesting that they are allochthonous and not actively used for FOS metabolism. A recent study found GH32 enzyme in *Bacillus subtilis* phages.^52^

Four strains in group N did not metabolize any of the oligosaccharides tested. These results are consistent with their GH profiles, as they lack proteins assigned to GH8, GH32, or GH36. These microbes were thus not directly activated by administration of the oligosaccharides tested. As some of the butyrate producers, including *A. hallii*, can obtain energy by a combination of acetate and lactate and produce butyrate,^20^ indirect stimulation by the acids produced through the metabolism of the oligosaccharides in other gut microbes may be possible. *Butyricimonas* spp. in the phylum Bacteroidetes are members of this poor GH holder group, although a previous study reported abundant GH family proteins (mean of 130 proteins) in 29 Bacteroidetes species (belonging to the genera *Bacteroides, Parabacteroides*, and *Prevotella*) originating from the human gut microbiota.^53^ Bacteroidetes organisms are able to produce biomass in the absence of fermentable carbohydrates,^7,54^ and similar results were obtained in the present study (Figure 1). Oligosaccharide metabolic properties in butyrate producers, as determined in the present study, are slightly inconsistent with a previous study,^54^ e.g. FOS metabolism in *R. hominis* and XOS metabolism in *A. caccae*, although the same strains were used. The discrepancy may be due to different oligosaccharides used in the studies. *Roseburia hominis* lacks GH32 protein and *A. caccae* lacks GH8 proteins (Table 2), consistent with their *in vitro* metabolic properties observed in the present study.

Phylogenetic analysis of GH32 enzymes produced four major clusters. Of the four clusters, enzymes classified in clusters 1, 2, and 3 had FOS degradation activity with different specific activities, except for *A.butyraticus*-GH32-2. Genes encoding GH32 enzymes in cluster 2 found in *Anaerostipes* spp. were located adjacent to genes encoding the PTS in each genome, suggesting that their innate substrates are phosphorylated-sucrose and -FOSs. Therefore, they are classified as S6PH.^55^ S6PH catalyzes the hydrolysis of both phosphorylated- and non-phosphorylated-products, but the enzyme has a markedly different *Km* for sucrose-6-phosphate and sucrose, 0.28 mM and 40 mM, respectively, in *Fusobacterium mortiferum*.^56^ Due to the commercial unavailability of phosphorylated-FOSs, specific activities of the phosphorylated-products were unable to be assessed in the present study. Substrate specificity of phospho-α-glucosidase in *F. mortiferum* was not influenced by the presence of phosphorylation.^57,58^ Two of the three GH32 enzymes in cluster 3 (*A.butyraticus*-GH32-3 and *A.hadrus*-GH32-4) contained components of signal sequences and CBM66, i.e., they are extracellular enzymes. On the other hand, *A.butyraticus*-GH32-3 is divided into two proteins (Locus_Tag ANBU17_10420 and ANBU17_10430) because of a stop codon. An intracellular enzyme only possessing the catalytic domain of *A.butyraticus*-GH32-3 (Locus_Tag ANBU17_10420) and fused extracellular whole *A.butyraticus*-GH32-3 (Locus_Tag ANBU17_10420 and ANBU17_10430), whose stop codon (TAG) was replaced with a glutamine codon (CAG) according to the sequences of *Clostridium spiroforme* (accession no. WP_087286452), exhibited similar FOS degradation activities, and degraded more kestose and nystose than sucrose. Similar degradation activities are also shared with *A.hadrus*-GH32-4. CBM66 binds the terminal fructosides of fructans in exo-acting β-fructosidase of *Bacillus subtilis* and removal of the CBM66 component resulted in an approximately 100-fold reduction in activity against levan.^59^ Extracellular GH enzymes equipped with CBMs are advantageous to obtain nutrients efficiently under the competitive gut microbiota^49,60^ and are also involved in symbiosis with other members in the microbiota by providing degradants.^61,62^ Similar functions may be observed for *A.butyraticus*-GH32-3 and *A.hadrus*-GH32-4 equipped with CBM66. Genes encoding four GH32 enzymes in cluster 1 (*A.rectalis*-GH32-1, *F.prausnitzii*-GH32, *R.faecis*-GH32, and *R.inulinivorans*-GH32) and one in cluster 3 (*R.intestinalis*-GH32) are adjacent to ABC transporter, suggesting that they are β-fructofuranosidase/levanase/inulinase. GH32 enzymes in these clusters had similar relative degradation activities against the three substrates or higher activities with the longer DP carbohydrates. These activities are consistent with the characteristics of adjacent transporters because ABC transporters generally import longer DP carbohydrates.^63^ PTSs are usually size-restricted transporters,^63^ and they were located adjacent to genes encoding cluster 2 enzymes hydrolyzing short DP carbohydrates. *A.hadrus*-GH32-4 and *A.butyraticus*-GH32-3 in cluster 3 are extracellular enzymes hydrolyzing longer DP carbohydrates, and adjacent PTS may be used to transport short DP carbohydrates after extracellular hydrolysis of long DP carbohydrates. GH32 enzymes in cluster 4 were not active against any of the tested FOSs, arabinan, inulin, or levan, suggesting that they have different unknown substrates. These enzymes were classified as GH32 by CAZy assignment but do not have the conserved NDPNG motif, suggesting that they are not GH32 enzymes. Previous studies found that the NDPNG motif is more associated with hydrolase/transferase activity than specificity,^64^ and amino acid replacement in this region (replacement of the initial N to S in the NDPNG motif) resulted in significant reduction of activity of the bacterial GH32 enzyme.^65^ The present study found that the NDPNG motif is involved in hydrogen bonding with substrates in GH32 enzymes. Based on BLASTP analysis, GH32 enzymes in cluster 4 are related to GH43 enzymes (data not shown). These enzymes were only found in one active-FOS metabolizer (i.e. *A. hadrus*) and two inactive-metabolizers (i.e. *A. rectalis* and *C. eutactus*), whereas *A. hadrus* possesses three alternative active GH32 enzymes to hydrolyze FOSs. Therefore, the presence of GH32 enzymes in cluster 4 does not impact the growth of butyrate producers.

Eight amino acid residues each are involved in hydrogen bonds with β-fructofuranose in the reference *B.longum*-GH32 β-fructofuranosidase^33^ and *L.gasseri-*GH32 S6PH (Table 3). The eight amino acids in *B.longum*-GH32 were all conserved in GH32 enzymes in cluster 1. *B.longum*-GH32 degrades sucrose, kestose, and nystose well,^33^ and this activity is consistent with that recorded for GH32 enzymes in cluster 1 (Figure 5). GH32 enzymes in cluster 3 also had similar FOS degradation activity to *B.longum*-GH32; however, Ser114 in *B.longum*-GH32 was substituted with threonine or alanine. GH32 enzymes in cluster 2 exhibited degradation activity for sucrose and kestose, but it was low or absent with nystose, and these enzymes substituted Trp78 with glycine or histidine. Trp105 in GH32 β-fructofuranosidase of *Xanthophyllomyces dendrorhous*, an equivalent locus to Trp78 in *B.longum*-GH32, mainly accommodates binding with 6-kestose at subsite +2.^66^ This suggests that Trp78, but not Ser114, is among the key factors for the degradation activity of nystose. The eight amino acids involved in hydrogen bonds with β-fructofuranose of *L.gasseri-*GH32 were conserved in GH32 enzymes in cluster 2. Although specific activities of *L.gasseri-*GH32 and nystose metabolic properties of the host strain 224–1 have not been characterized, other strains of *L. gasseri* are known to metabolize kestose, but not nystose.^26,67^ Two or three of the eight amino acid residues involved in hydrogen bonds with β-fructofuranose of *B.longum*-GH32 (Asn53, Gln70, and Trp78) were substituted with other amino acids in GH32 enzymes of cluster 4. These amino acid replacements and lack of the NDPNG motif, as described above, would result in no activity against FOSs.

A previous study reported that most genes encoding GH formed PULs with genes encoding transporters and regulators in the genomes of *Roseburia* spp. and *A. rectalis*.^34^ The study defined PUL as being a locus encoding, at minimum, one polysaccharide-degrading enzyme, a carbohydrate transport system, and a transcriptional regulator. Based on this definition, of the 17 GH32 proteins found in the present study, genes encoding 12 proteins formed 11 PULs, and *A.hadrus*-GH32-2 and *A.hadrus*-GH32-3 were located in a single PUL (Figure 6). Genes encoding carbohydrate kinase were found in seven of the 11 PULs. The number of genes encoding transporters and regulators, and arrangements of the genes in the 11 PULs are highly divergent among PULs, whereas relatively similar PUL structures were reported for GH32 proteins of butyrate producers in a previous study.^34^ Most PULs containing genes encoding GH32 enzymes found in the present study contain a single GH enzyme, whereas those for other GHs in butyrate producers contain multiple genes encoding several GHs, up to seven genes.^34^

In conclusion, 14 gut butyrate producers exhibited different FOS metabolic properties among organisms. Profiles of GH storage suggested diverse polysaccharide metabolic properties in the strains. Phylogenetic analysis separated GH32 enzymes found in butyrate producers into four clusters, and the enzymes generally had similar degradation activities among the clusters. GH32 enzymes exhibiting FOS degradation activities were conserved in all six strains metabolizing FOS and in three of the eight strains that did not metabolize FOS, suggesting that GH32 enzymes in the three strains are not actively used in metabolism. The present study highlighted that even if functional genes are present in microbes, they are sometimes unable to metabolize the substrates. This should be carefully considered in metagenomic studies to understand the metabolic potential of gut microbiota. The present study sheds light on the important characteristics of GH32 enzymes and their relationship with metabolic properties in important health-related microbes to discuss the potential of FOS as prebiotics.

## Materials and methods

### Bacterial strains and pre-culturing

Fourteen strains of gut butyrate-producing bacteria were used in the present study (Table 1). These strains were obtained from the Japan Collection of Microorganisms (JCM) and the American Type Culture Collection (ATCC). They included the predominant gut butyrate-producing bacteria in humans, i.e. *F. prausnitzii* in the *Clostridium* cluster IV and *Roseburia* spp., *A. rectalis, A. hallii* and *Anaerostipes* spp. in the *Clostridium* cluster XIVa.^13,68^ A few more human gut butyrate producers belonging to the *Clostridium* clusters IV and XIVa, and the phylum Bacteroidetes were also included to assess diverse metabolic characteristics in butyrate-producing bacteria. Basal YCFA broth supplemented with 0.5% (w/v) glucose was purged with N_2_ gas using an O_2_-removal unit (Model AG-2, Sanshin, Kanagawa, Japan) and used for pre-culturing. The composition of YCFA broth was described elsewhere.^69^ Bacterial cells were inoculated into the broth by injection and cultured at 37°C for 24 or 48 h.

### Growth on oligosaccharides

To study FOS metabolic properties, kestose (DP3; B Food Science, Japan), nystose (DP4; Wako Chemical, Japan), and a FOS mixture (FOSs, containing DP3-5; Wako Chemical) were used. XOSs (mainly DP2-5; B Food Science) and raffinose (Wako Chemical), which were reported as bifidogenic prebiotics, were also included in the present study. The composition of carbohydrates was described previously,^7^ and the purity of kestose, nystose, and raffinose was higher than 98% (w/w). Basal YCFA broth supplemented with 0.5% (w/v) oligosaccharide was used to study oligosaccharide metabolic properties of butyrate-producing bacteria. Sugar-free YCFA broth and YCFA broth supplemented with 0.5% glucose were included as controls. These tested broths were also purged with N_2_ gas to be oxygen-free. Twenty microliters of the pre-cultured cells were inoculated into 2 ml of the tested broth and incubated anaerobically at 37°C for 72 h. Growth was monitored at 660 nm using a spectrophotometer (model U-2800A, Hitachi, Japan) every 24 h. This experiment was performed in triplicate.

### Butyrate production and metabolic profiles of oligosaccharides

Butyrate produced by the metabolism of oligosaccharides was measured by high-performance liquid chromatography (HPLC) combined with an Aminex HPX- 87 H column (Bio-Rad, Japan), and the oligosaccharide composition of FOSs and XOSs before/after culturing was determined using high-performance anion exchange chromatography coupled with a pulsed amperometric detection (HPAEC-PAD) system (model ICS-3000, Dionex, United Kingdom) and a Dionex CarboPac PA1 column (Thermo Scientific, Japan), as described previously.^26^ These were determined in culture supernatants after incubation of 72 h. These experiments were performed in triplicate.

### Genome analysis and identification of GH32 enzymes

Of the 14 strains used in the *in vitro* oligosaccharide metabolic test, draft or complete genomes of ten strains were obtained from the GenBank, RefSeq, or the JGI Genome Portal^70^ database and used in the analysis. Genomes of the remaining four strains, which were *A. butyraticus* JCM 17466 ^T^, *Butyricicoccus faecihominis* JCM 31056 ^T^, *A. hallii* JCM 31263, and *C. eutactus* JCM 31265, were sequenced by the Illumina platform. Genomic DNA was isolated from cells as described previously.^71^ Assembly and annotation of the sequences, and quality check of the resulting genomic data were conducted by methods described previously.^72^ The genomic data were used to search for GH family enzymes using dbCAN2 in the CAZy database with HMMER, DIAMOND, and Hotpep tools.^73^ GH proteins were identified when detected by two of the three tools, as recommended by the database.^73^ Numbers of estimated proteins in each GH family of the strains were used to prepare a dendrogram using the hclust function with the Ward.D2 algorithm in the R package (version 3.6.2). The signal peptide in GH proteins was identified by CAZy and SignalP-5.0 programs.^74^ The phylogenetic tree was constructed using the program ClustalW, version 2.1.^75^ The number of bootstrapping replicates was 1,000.

To select amino acid residues that are essential for substrate specificity, the structures of two GH32 enzymes, *B.longum*-GH32 β-fructofuranosidase (PDB ID, 3PIJ) and *L.gasseri*-GH32 S6PH (PDB ID, 6NU8), were obtained. *B.longum*-GH32 was reported to hydrolyze sucrose, kestose, and nystose,^33^ whereas *L.gasseri*-GH32 is the only bacterial S6PH whose structure is currently available. Eight amino acid residues forming hydrogen bonds with β-fructofuranose at subsite −1 in *B.longum*-GH32 were obtained from elsewhere,^33^ and those in *L.gasseri*-GH32 were determined using the crystal structure of the enzyme complexed with β-fructofuranose (PDB ID 6NU7) and PyMOL software (version 2.4; https://pymol.org/2/) with the default setting (within 3.3 Å). GH32 enzymes found in 14 butyrate producers were aligned with the two reference GH32 enzymes described above, and amino acid residues at positions equivalent to the hydrogen bonds with β-fructofuranose in the two reference enzymes were compared among clusters.

### *Activity of recombinant GH32 enzymes expressed in* E. coli

Genomic DNA was isolated from cells of the butyrate producers by a previously described method^71^ and used as templates to amplify genes encoding GH32 enzymes. Plasmids were constructed using the In-Fusion Cloning kit (Takara-bio, Japan). Plasmids and the genes encoding GH32 enzymes were amplified using the KOD Plus DNA polymerase kit (Toyobo, Japan) combined with primers listed in Supplemental Table S2, and fused using the In-Fusion Cloning kit according to the manufacturer’s instructions. pET28a (Merck, Germany) was used for cloning of intracellular GH32 enzymes and pCDF-PgsA^76^ was used for extracellular GH32 enzymes. The latter plasmid was developed to study the activities of extracellular GH32 enzymes using the surface display system on *E. coli* cells.^76^ *Escherichia coli* JM109 (Takara-bio, Japan) were transformed with the fused plasmids, which were extracted from the transformed strains using the FastGene Plasmid Mini Kit (Nippon Genetics, Japan) and used to transform *E. coli* BL21 (DE3, Takara-bio). Transformed *E. coli* strains were cultured in Terrific broth (Sigma-Aldrich, Japan) supplemented with 25 μg/ml of kanamycin sulfate (for pET28a-derivative plasmids) or 12.5 μg/ml of streptomycin sulfate (for pCDF-PgsA -derivative plasmids) at 20°C or 24°C for 24 h. The transformed *E. coli* BL21 (DE3) cells were cultured in Overnight Express™ Instant TB Medium (Merck, Germany) supplemented with antibiotics at 20°C for 24 h. For intracellular enzymes, preparation of cell-free extracts, enzyme assays, and carbohydrate profiles after enzyme reactions were performed as described previously.^32^ For extracellular enzymes, overnight-cultured recombinant *E.coli* cells were collected by centrifugation, washed, re-suspended in solution containing 30% substrates, and used to measure substrate degradation activities, as described elsewhere.^76^ Sucrose, kestose, and nystose were used as substrates for this assay, and arabinan, inulin, and levan were also included for GH32 enzymes of cluster 4.

For cloning of *A.butyraticus*-GH32-3, two *E. coli* recombinants were generated. One recombinant produced intracellular partial *A.butyraticus*-GH32-3 (Locus_Tag ANBU_10420) and the other produced extracellular whole *A.butyraticus*-GH32-3 (Locus_Tag ANBU_10420 and 10430). To prepare the extracellular *A.butyraticus*-GH32-3, genes encoding whole *A.butyraticus*-GH32-3 (Locus_Tag ANBU_10420 and 10430) were amplified by the method described above. The amplified product was fused with pCDF-PgsA (pCDF_PgsA_AB_LamG_Pre), as described above. pCDF_PgsA_AB_LamG_Pre was amplified with the primer pair of AB_LamG_fusion(Q) and AB_LamG_fusion(Q)_R for linearization and replacement of the stop codon (TAG) with a glutamine codon (CAG) according to the sequence of GH32 β-fructofuranosidase of *Clostridium spiroforme* (accession no. WP_087286452), and self-ligated using T4 Polynucleotide Kinase (Toyobo, Japan) and DNA Ligation Kit Ver.2.1 (Takara-bio, Japan). The GH32 β-fructofuranosidase of *Clostridium spiroforme* (accession no. WP_087286452) had the highest similarity (63%) with *A.butyraticus*-GH32-3 by BLAST analysis (data not shown). Activities of the extracellular *A.butyraticus*-GH32-3 (Locus_Tag ANBU_10420 and 10430) enzyme were studied using the surface display system on *E. coli* cells, and those of the intracellular partial *A.butyraticus*-GH32-3 (Locus_Tag ANBU_10420) were determined by preparing cell-free extracts, as described above.

## Supplementary Material

Supplemental Material
