# Supplementary material for: Characterization of fructooligosaccharide metabolism and fructooligosaccharide-degrading enzymes in human commensal butyrate producers
Source: Gut Microbes. 2021 Jan 13;13(1):1869503. doi: 10.1080/19490976.2020.1869503 (PMC7833758; doi:10.1080/19490976.2020.1869503)
Supplement: Supplemental Material [file KGMI_A_1869503_SM8939.zip › SUPPLEMENT/supplemental Fig S1.pptx]

## Slide 1
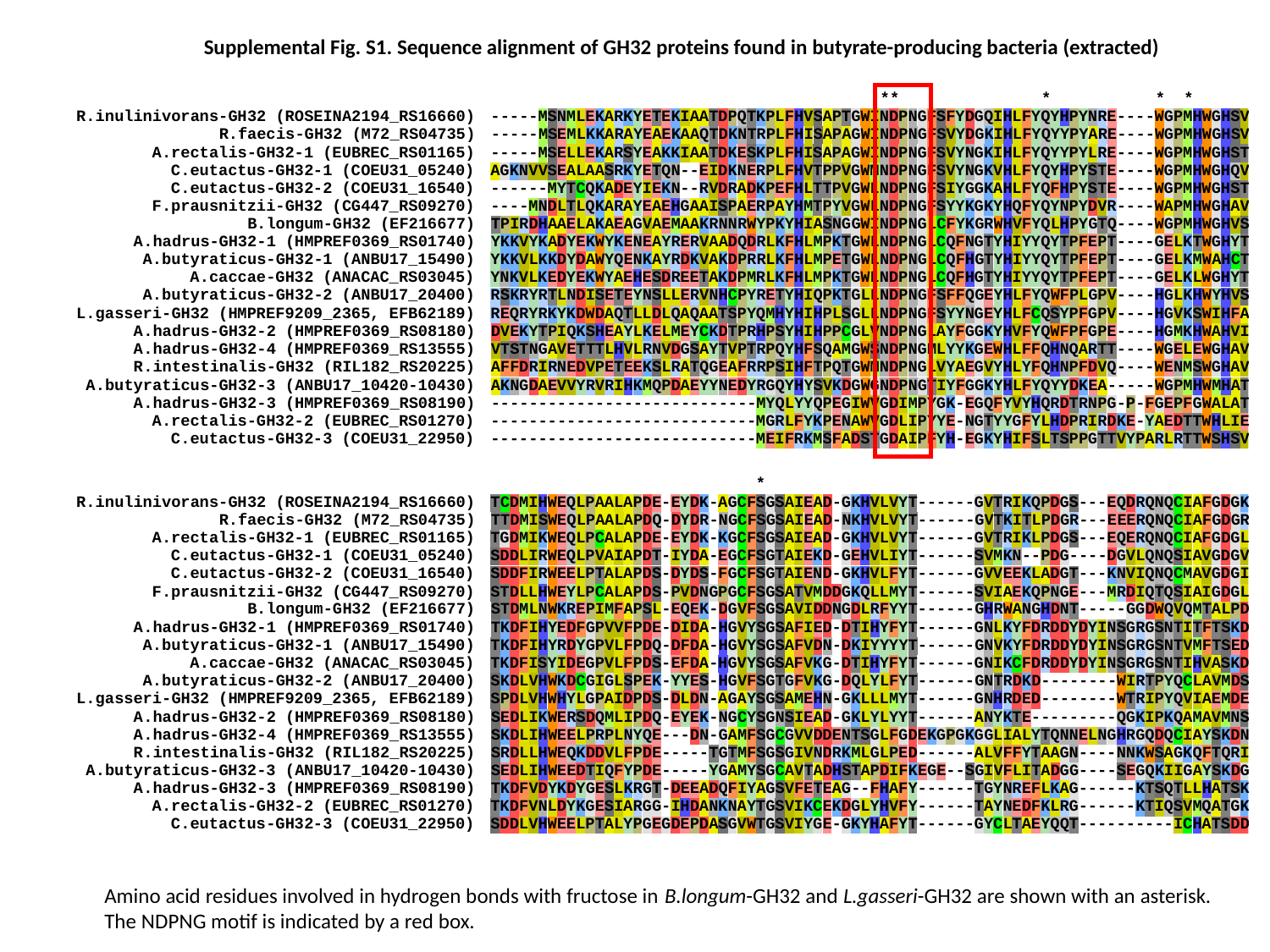

Supplemental Fig. S1. Sequence alignment of GH32 proteins found in butyrate-producing bacteria (extracted)
Amino acid residues involved in hydrogen bonds with fructose in B.longum-GH32 and L.gasseri-GH32 are shown with an asterisk.
The NDPNG motif is indicated by a red box.

## Slide 2
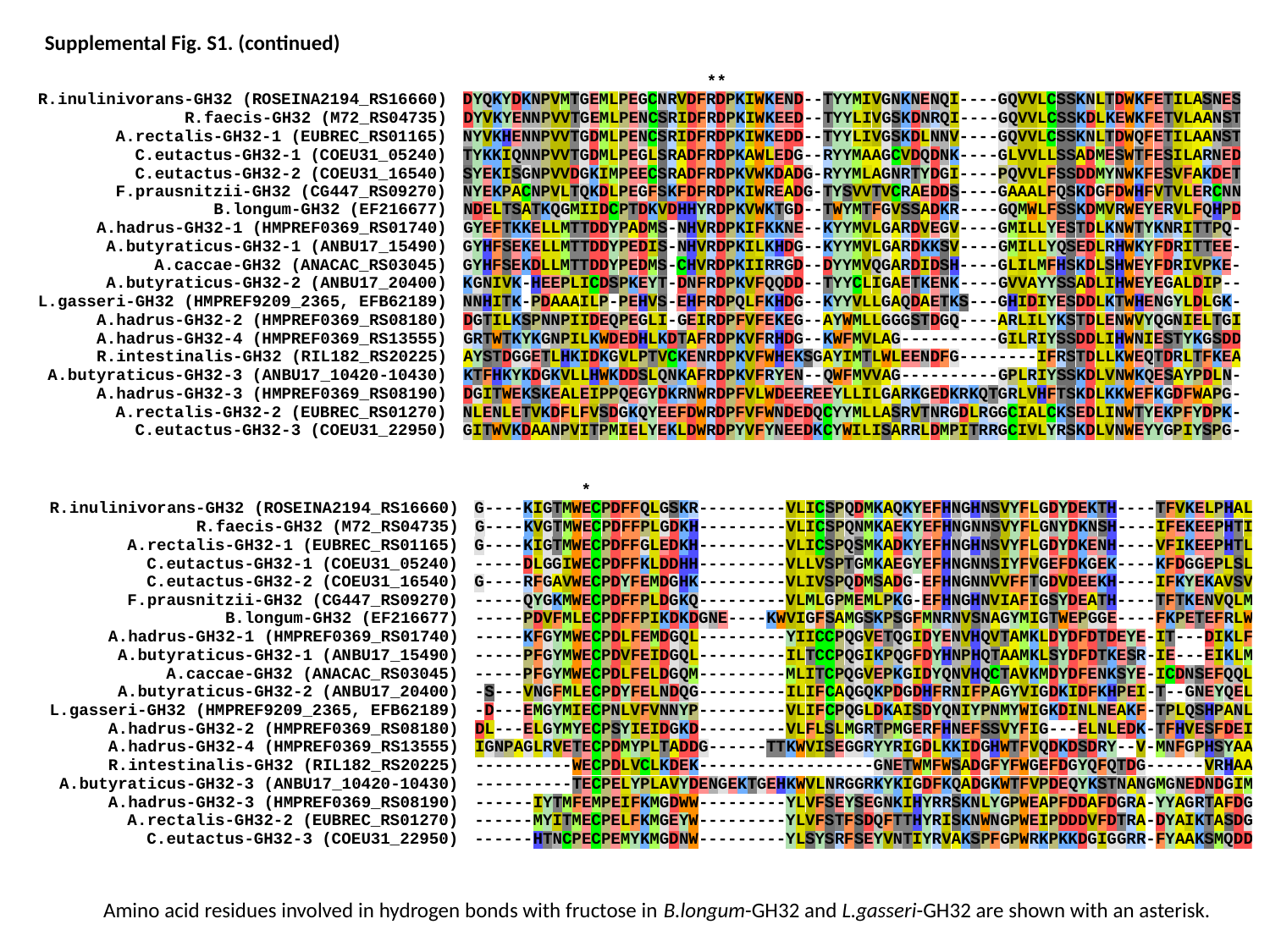

Supplemental Fig. S1. (continued)
Amino acid residues involved in hydrogen bonds with fructose in B.longum-GH32 and L.gasseri-GH32 are shown with an asterisk.
