# Supplementary material for: Characterization of fructooligosaccharide metabolism and fructooligosaccharide-degrading enzymes in human commensal butyrate producers
Source: Gut Microbes. 2021 Jan 13;13(1):1869503. doi: 10.1080/19490976.2020.1869503 (PMC7833758; doi:10.1080/19490976.2020.1869503)
Supplement: Supplemental Material [file KGMI_A_1869503_SM8939.zip › SUPPLEMENT/supplemental Table S2.pptx]

## Slide 1
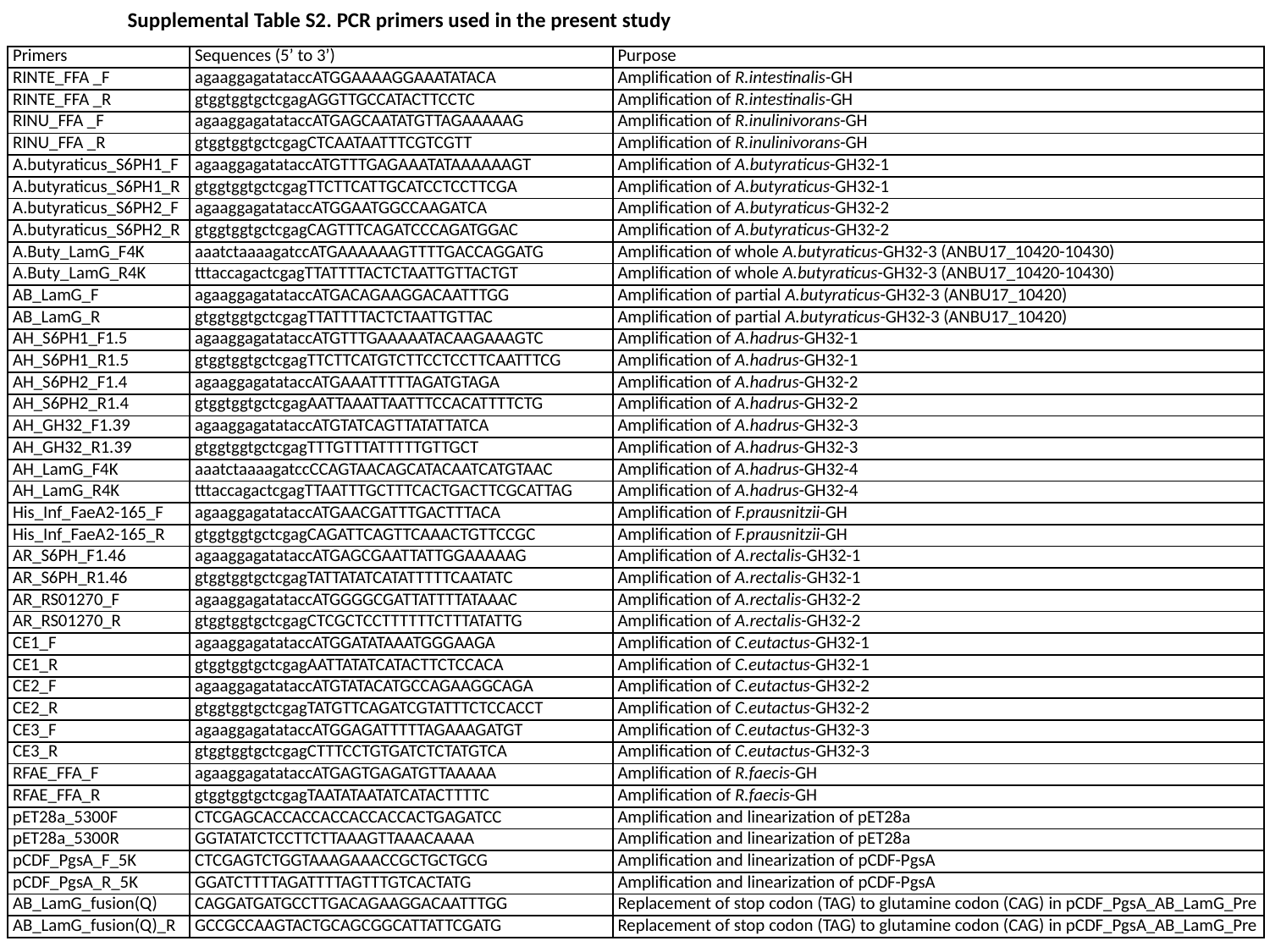

Supplemental Table S2. PCR primers used in the present study
| Primers | Sequences (5’ to 3’) | Purpose |
| --- | --- | --- |
| RINTE\_FFA \_F | agaaggagatataccATGGAAAAGGAAATATACA | Amplification of R.intestinalis-GH |
| RINTE\_FFA \_R | gtggtggtgctcgagAGGTTGCCATACTTCCTC | Amplification of R.intestinalis-GH |
| RINU\_FFA \_F | agaaggagatataccATGAGCAATATGTTAGAAAAAG | Amplification of R.inulinivorans-GH |
| RINU\_FFA \_R | gtggtggtgctcgagCTCAATAATTTCGTCGTT | Amplification of R.inulinivorans-GH |
| A.butyraticus\_S6PH1\_F | agaaggagatataccATGTTTGAGAAATATAAAAAAGT | Amplification of A.butyraticus-GH32-1 |
| A.butyraticus\_S6PH1\_R | gtggtggtgctcgagTTCTTCATTGCATCCTCCTTCGA | Amplification of A.butyraticus-GH32-1 |
| A.butyraticus\_S6PH2\_F | agaaggagatataccATGGAATGGCCAAGATCA | Amplification of A.butyraticus-GH32-2 |
| A.butyraticus\_S6PH2\_R | gtggtggtgctcgagCAGTTTCAGATCCCAGATGGAC | Amplification of A.butyraticus-GH32-2 |
| A.Buty\_LamG\_F4K | aaatctaaaagatccATGAAAAAAGTTTTGACCAGGATG | Amplification of whole A.butyraticus-GH32-3 (ANBU17\_10420-10430) |
| A.Buty\_LamG\_R4K | tttaccagactcgagTTATTTTACTCTAATTGTTACTGT | Amplification of whole A.butyraticus-GH32-3 (ANBU17\_10420-10430) |
| AB\_LamG\_F | agaaggagatataccATGACAGAAGGACAATTTGG | Amplification of partial A.butyraticus-GH32-3 (ANBU17\_10420) |
| AB\_LamG\_R | gtggtggtgctcgagTTATTTTACTCTAATTGTTAC | Amplification of partial A.butyraticus-GH32-3 (ANBU17\_10420) |
| AH\_S6PH1\_F1.5 | agaaggagatataccATGTTTGAAAAATACAAGAAAGTC | Amplification of A.hadrus-GH32-1 |
| AH\_S6PH1\_R1.5 | gtggtggtgctcgagTTCTTCATGTCTTCCTCCTTCAATTTCG | Amplification of A.hadrus-GH32-1 |
| AH\_S6PH2\_F1.4 | agaaggagatataccATGAAATTTTTAGATGTAGA | Amplification of A.hadrus-GH32-2 |
| AH\_S6PH2\_R1.4 | gtggtggtgctcgagAATTAAATTAATTTCCACATTTTCTG | Amplification of A.hadrus-GH32-2 |
| AH\_GH32\_F1.39 | agaaggagatataccATGTATCAGTTATATTATCA | Amplification of A.hadrus-GH32-3 |
| AH\_GH32\_R1.39 | gtggtggtgctcgagTTTGTTTATTTTTGTTGCT | Amplification of A.hadrus-GH32-3 |
| AH\_LamG\_F4K | aaatctaaaagatccCCAGTAACAGCATACAATCATGTAAC | Amplification of A.hadrus-GH32-4 |
| AH\_LamG\_R4K | tttaccagactcgagTTAATTTGCTTTCACTGACTTCGCATTAG | Amplification of A.hadrus-GH32-4 |
| His\_Inf\_FaeA2-165\_F | agaaggagatataccATGAACGATTTGACTTTACA | Amplification of F.prausnitzii-GH |
| His\_Inf\_FaeA2-165\_R | gtggtggtgctcgagCAGATTCAGTTCAAACTGTTCCGC | Amplification of F.prausnitzii-GH |
| AR\_S6PH\_F1.46 | agaaggagatataccATGAGCGAATTATTGGAAAAAG | Amplification of A.rectalis-GH32-1 |
| AR\_S6PH\_R1.46 | gtggtggtgctcgagTATTATATCATATTTTTCAATATC | Amplification of A.rectalis-GH32-1 |
| AR\_RS01270\_F | agaaggagatataccATGGGGCGATTATTTTATAAAC | Amplification of A.rectalis-GH32-2 |
| AR\_RS01270\_R | gtggtggtgctcgagCTCGCTCCTTTTTTCTTTATATTG | Amplification of A.rectalis-GH32-2 |
| CE1\_F | agaaggagatataccATGGATATAAATGGGAAGA | Amplification of C.eutactus-GH32-1 |
| CE1\_R | gtggtggtgctcgagAATTATATCATACTTCTCCACA | Amplification of C.eutactus-GH32-1 |
| CE2\_F | agaaggagatataccATGTATACATGCCAGAAGGCAGA | Amplification of C.eutactus-GH32-2 |
| CE2\_R | gtggtggtgctcgagTATGTTCAGATCGTATTTCTCCACCT | Amplification of C.eutactus-GH32-2 |
| CE3\_F | agaaggagatataccATGGAGATTTTTAGAAAGATGT | Amplification of C.eutactus-GH32-3 |
| CE3\_R | gtggtggtgctcgagCTTTCCTGTGATCTCTATGTCA | Amplification of C.eutactus-GH32-3 |
| RFAE\_FFA\_F | agaaggagatataccATGAGTGAGATGTTAAAAA | Amplification of R.faecis-GH |
| RFAE\_FFA\_R | gtggtggtgctcgagTAATATAATATCATACTTTTC | Amplification of R.faecis-GH |
| pET28a\_5300F | CTCGAGCACCACCACCACCACCACTGAGATCC | Amplification and linearization of pET28a |
| pET28a\_5300R | GGTATATCTCCTTCTTAAAGTTAAACAAAA | Amplification and linearization of pET28a |
| pCDF\_PgsA\_F\_5K | CTCGAGTCTGGTAAAGAAACCGCTGCTGCG | Amplification and linearization of pCDF-PgsA |
| pCDF\_PgsA\_R\_5K | GGATCTTTTAGATTTTAGTTTGTCACTATG | Amplification and linearization of pCDF-PgsA |
| AB\_LamG\_fusion(Q) | CAGGATGATGCCTTGACAGAAGGACAATTTGG | Replacement of stop codon (TAG) to glutamine codon (CAG) in pCDF\_PgsA\_AB\_LamG\_Pre |
| AB\_LamG\_fusion(Q)\_R | GCCGCCAAGTACTGCAGCGGCATTATTCGATG | Replacement of stop codon (TAG) to glutamine codon (CAG) in pCDF\_PgsA\_AB\_LamG\_Pre |
